# When is Genomic Testing Cost-Effective? Testing for Lynch Syndrome in Patients with Newly-Diagnosed Colorectal Cancer and Their Relatives

**DOI:** 10.3390/healthcare3040860

**Published:** 2015-09-24

**Authors:** Scott D. Grosse

**Affiliations:** National Center on Birth Defects and Developmental Disabilities, Centers for Disease Control and Prevention, Atlanta, GA 30341, USA; E-Mail: sgrosse@cdc.gov; Tel.: +404-498-3074

**Keywords:** health economics, cost-effectiveness, genomics, genetic testing, hereditary cancer, Lynch syndrome, colorectal cancer

## Abstract

Varying estimates of the cost-effectiveness of genomic testing applications can reflect differences in study questions, settings, methods and assumptions. This review compares recently published cost-effectiveness analyses of testing strategies for Lynch Syndrome (LS) in tumors from patients newly diagnosed with colorectal cancer (CRC) for either all adult patients or patients up to age 70 along with cascade testing of relatives of probands. Seven studies published from 2010 through 2015 were identified and summarized. Five studies analyzed the universal offer of testing to adult patients with CRC and two others analyzed testing patients up to age 70; all except one reported incremental cost-effectiveness ratios (ICERs) < $ 100,000 per life-year or quality-adjusted life-year gained. Three studies found lower ICERs for selective testing strategies using family history-based predictive models compared with universal testing. However, those calculations were based on estimates of sensitivity of predictive models derived from research studies, and it is unclear how sensitive such models are in routine clinical practice. Key model parameters that are influential in ICER estimates included 1) the number of first-degree relatives tested per proband identified with LS and 2) the cost of gene sequencing. Others include the frequency of intensive colonoscopic surveillance, the cost of colonoscopy, and the inclusion of extracolonic surveillance and prevention options.

## 1. Introduction

With increasing translation of genetic testing to clinical practice, the cost-effectiveness of clinical applications of molecular genetic tests has become a “hot” question in health economics and genomics. Recent systematic reviews have examined “genetic testing technologies” [1], “genomic technologies” [2], “personalized medicine” [3], and “individualized medicine” [4]. Hatz *et* *al*. encourage researchers to seek to understand “how” or “when” rather than “whether” genetic testing is cost-effective [4]. That requires researchers to address the heterogeneity in cost-effectiveness estimates for the same genetic test in order to identify influential contextual factors, which is rarely done.

A precondition for the demonstration of cost-effectiveness is evidence of effectiveness, *i*.*e*., the ability to prevent mortality and morbidity. As a recent commentary on economic evaluation in genomic medicine put it, evidence of clinical utility (effectiveness) is needed before cost-utility or cost-effectiveness can be shown [5]. Public health genomics focuses on promoting the implementation of evidence-based genetic testing applications that have been shown to have health impact. In particular, the US Centers for Disease Control and Prevention (CDC) in 2012 began a process for identifying Tier 1 genomic applications that have a synthesized evidence base supporting implementation [6].

One of the most prominent Tier 1 public health genomic applications is testing for Lynch Syndrome (LS) in tumor specimens from patients newly diagnosed with colorectal cancer (CRC) using preliminary testing using either immunohistochemistry (IHC) or microsatellite instability (MSI), followed by genetic sequencing and deletion testing to identify a mutation on an MMR gene [7,8,9]. Lynch Syndrome (LS) is an autosomal dominant cancer syndrome associated with a very high risk of both CRC and endometrial cancer (EC) [10]. Testing for LS in adults with CRC permits cascade testing of relatives of probands. Relatives who are found to have LS and agree to undergo intensive surveillance for CRC through colonoscopy every 1–2 years can substantially reduce the risks of: developing CRC, an advanced stage tumor if cancer does occur, and the likelihood of death from CRC. In addition, women who are found to have LS may take action to reduce the risk of developing EC. Most of the benefit from identification of LS consists of the gains in life expectancy among relatives identified through cascade testing.

Testing of patients with CRC for LS can be either selective or universal. Traditionally, patients have been selected for testing based on either Amsterdam II criteria [11] or Revised Bethesda Guidelines (RBG) criteria [12]. The RBG criteria combine information on age of cancer diagnosis, tumor type, and stringent family history criteria suggestive of LS. Such criteria in a research setting are highly sensitive in identifying individuals with LS [13]. In some countries, RBG criteria are considered standard care [14]. Complex statistical models which integrate age at onset, tumor characteristics and detailed family history of LS-related cancers, can also be used [15]. The limitation to selective testing is that collecting, analyzing, interpreting, and appropriately using the information to guide testing is challenging. In practice few LS patients may be identified through the use of RBG criteria [16,17].

Universal testing for LS in adults with newly diagnosed CRC was first recommended in 2009 by the CDC sponsored Evaluation of Genomics in Practice and Prevention (EGAPP) working group [18,19,20]. Universal testing was subsequently endorsed by other US groups [17,21,22]. Testing can also be targeted to CRC patients based on age cutoffs such as 60 [23] or 70 years [24] or combination of universal testing below an age cutoff of 70 and selective testing in patients over age 70 who meet RBG criteria [25,26].

We explore under what assumptions universal or near-universal tumor testing for LS in patients with CRC followed by cascade testing of relatives of those found to have LS is likely to be cost-effective in terms of promoting survival or quality-adjusted survival. Specifically, we assessed estimates from “full” CEAs that calculate health outcomes to address the following questions: (1) how does universal testing for LS compare with selective or targeted testing including use of RBG criteria or statistical models; (2) how do different age cutoffs for targeted testing affect the incremental cost-effectiveness of universal testing relative to targeted testing; and (3) to what extent do differences in epidemiologic assumptions account for differences in estimates between studies of the incremental cost-effectiveness ratio (ICER) of testing for LS? Previous discussions of differences in ICER estimates of testing for LS have either touched lightly on epidemiological parameters or been limited to comparisons of pairs of studies [14,27].

## 2. Methods

### Identification of Relevant Studies

In this paper, we reviewed full CEAs of genetic testing for LS in tumor tissues of patients with newly diagnosed CRC followed by cascade testing of relatives. Specifically, CEA studies were included if they reported estimates of health outcomes using the metrics of either discounted life-years saved (LYs) or quality-adjusted life-years (QALYs) gained as the denominator of ICERs. We excluded “partial” CEAs which reported estimates of cost per case detected but not cost per unit of health gains because knowing that one strategy is cheaper than another does not provide information on the value of either intervention [21,28].

We included analyses published after 2009 in which at least one strategy involved either universal testing of unselected CRC patients or near-universal testing of patients up to age 70 years. The starting date of 2009 was chosen because the objective was to assess estimates of cost-effectiveness published since the EGAPP Working Group issued its recommendation of universal LS testing in 2009 [18] and the evidence review supporting that recommendation was published the same year [19].

Articles were identified through one of two ways. First, articles included in a systematic review of economic evaluation of LS testing published in 2014 [29] that met our inclusion criteria were included. Second, we conducted a search in PubMed on March 29, 2015 using “cost-effectiveness” and “Lynch syndrome” as search terms in all fields to identify relevant articles published since the end date (2012) used in the previous review; no language restriction was imposed. In addition, experts on LS testing were contacted and unrestricted internet searches were conducted; no other studies that met the inclusion criteria were identified. Each CEA reported both base case model estimates, which are point estimates of ICERs incorporating the assumptions that are considered most likely, and sensitivity analyses that take into account uncertainty in model parameters. In this paper we focused on the base case estimates from each study, although we discuss some of the findings of sensitivity analyses. We summarize ICERs for the lowest-cost laboratory testing strategy for each population testing strategy (universal or targeted) from each cited study. To assure comparability of ICER estimates across studies, this study used 2014 US dollars as a standard currency year. Published estimates in other currencies were converted to US dollars using the exchange rate for the original currency year. Estimates in US dollars from different years were converted to 2014 values using the US gross domestic product (GDP) implicit price deflator to adjust for changes in the purchasing power of the US dollar for studies that reported the currency year for their cost assumptions.

## 3. Results and Discussion

### 3.1. Overview of Included Studies and Assumptions

Seven CEAs of routine genetic testing for LS in newly diagnosed CRC patients and subsequent cascade testing met the inclusion criteria. Four publications modeled testing for LS in the US healthcare context [30,31,32,33] (Table 1). One of the US studies was conducted at the CDC [32]. Two US articles reported different versions of a single CEA model developed at the University of California at San Francisco (UCSF), one with results in terms of LYs [31] and a subsequent article extending the model to project QALY gains [33]. A recent US publication reported an analysis conducted at the University of Southern California [30]. The remaining three studies modeled testing in the Netherlands, the United Kingdom, and Germany, conducted respectively by the Radboud University Medical Center in Nijmegen [34], PenTAG in Exeter [29,35], and the Helmholtz Center in Munich [14]. Of the seven CEA articles, one did not define the study perspective [34], and the remaining studies referenced the healthcare sector [32], societal [30], or third-party payer [14,29,31,33] perspectives. All studies discounted costs and health outcomes in future years using the same annual discount rate, varying from 3% to 4% depending on standard practice in the country in which each study was conducted.

The studies included in this review also differed in terms of assumptions as to what interventions follow from identification of LS mutation carriers among probands and asymptomatic relatives (Table 1). One CEA study assumed that a small percentage of mutation carriers (3%) would choose to undergo subtotal colectomy to reduce the risk of developing CRC [31] in addition to assumptions about the impact of LS diagnoses on the probability of risk-reducing strategies in patients with CRC [29,31]. However, expert guidelines do not encourage prophylactic colectomy in healthy mutation carriers [25].

Two studies assumed that female mutation carriers would be offered testing or prophylactic surgery to prevent gynecologic cancers associated with LS. Prophylactic total abdominal hysterectomy and bilateral salpingo-oophorectomy (TAH/BSO) have been shown in observational data to prevent the subsequent occurrence of ovarian and endometrial cancers in women with LS [36], and such surgery is recommended to be offered to women who have completed childbearing [22,25]. In addition, women with LS can be offered annual surveillance for endometrial and ovarian cancer although it has not yet been shown that such screening is effective in reducing the risk of cancer [10,22]. Ladabaum *et* *al*. assumed that female mutation carriers would be offered annual screening with transvaginal ultrasonography and endometrial sampling starting at age 35 years and TAH/BSO at age 40 years [31]. The study modeled the costs of surveillance, but assumed no health outcomes, whether benefits or harms [31]. The UK PenTAG group modeled the offer of TAH/BSO at a minimum age of 45 [29].

Prophylactic aspirin has been shown to reduce the risk of CRC in individuals with LS [10,22,25]. The CAPP2 randomized trial demonstrated a roughly 40% reduction in CRC incidence among subjects randomized to receive daily aspirin prophylaxis and a roughly 60% reduction among subjects who adhered to the protocol [37]. The German Helmholtz study was the first to model the use of prophylactic aspirin for the prevention of CRC in addition to intensive surveillance for CRC, conservatively assuming a 37% reduction in CRC for a limited number of years [14].

**Table 1:** Cost-effectiveness studies of testing strategies for Lynch Syndrome in patients with colorectal cancer.

Study	Country	Analytic Perspective	Discount Rate (per Annum) ^	Colonoscopic Surveillance Frequency	Other Preventive Strategies Modeled
Mvundura *et* *al*. [32]	USA	US healthcare system	3%	Every 2 years starting at 20 years	None
Ladabaum *et* *al*. [31] & Wang *et* *al*. [33]	USA	Third-party payer	3.5%	Every year starting at 25 years	Subtotal colectomy by mutation carriers TAH/BSO at age 40 years
Sie *et* *al*. [34]	Netherlands	Not stated	4%	Every 2 years	None
Snowsill *et* *al*. [29,35]	UK	UK National Health Service		Every 2 years	TAH/BSO at minimum age 45 years
Severin *et* *al*. [14]	Germany	German Statutory Health Insurance system	3%	Every year starting at 25 years	Aspirin prophylaxis
Barzi *et* *al*. [30]	USA	Societal	3%	Every year starting at 20 years	None

^ All studies applied the same discount rate to costs and health outcomes in future years. TAH/BSO: total abdominal hysterectomy and bilateral salpingo-oophorectomy.

### 3.2. Results—Incremental Cost-Effectiveness Ratios (ICERs)

Published estimates of ICERs of testing patients with CRC for LS along with testing relatives of probands vary. In Table 2, we reported non-negative ICERs for universal testing, age-targeted testing, and family history-targeted testing strategies. The Radboud study from the Netherlands [34] reported that LS testing appears to be dominant or cost-saving. In contrast, the Helmholtz study from Germany estimated that LS testing yields slight health improvements at high cost, with the lowest-cost strategy costing $ 106,000 per LY gained, and the ICER for universal testing was almost $ 350,000 per LY gained relative to targeted testing [14]. Two US studies that assessed the cost-effectiveness of targeted testing relative to both no testing and universal testing found ICERs about one-third as high as in the German study, <$ 35,000 and $ 120,000–150,000 per LY, respectively [30,31].

The base case ICERs of universal testing *vs*. no testing for the CDC study was approximately $ 25,000 per LY or $ 30,000 per QALY [32]. The authors of the CDC analysis recently reported updated results of the model, with a revised ICER (in 2014 USD) for universal testing of approximately $ 35,000 per LY relative to no testing [27]. The comparable results for other US studies are $ 39,000 to $ 45,000 per LY [30,31] or $ 64,000 per QALY [33].

### 3.3. Factors Contributing to Differences in Cost-Effectiveness Findings

The following subsections detail differences between studies that contribute to the variations in estimates of costs and effectiveness. For example, Severin *et* *al*. attribute the differences between their ICER estimates and those of previous studies in large part to differences between countries in reimbursement rates for genetic tests, the numbers of first-degree relatives (FDRs) available to be tested, and the willingness of people, both probands and FDRs, to undergo mutation testing [14].

**Table 2:** Base case incremental cost-effectiveness ratios of testing strategies for Lynch Syndrome in patients with colorectal cancer, adjusted to 2014 US dollars.

Study	Country	Strategy	Comparator	ICER (Nearest 100 US Dollars)	
				Per LY saved	Per QALY gained
**Universal *vs*. No Testing**					
Mvundura *et* *al*. [32] & Grosse *et* *al*. [27]	USA			$ 25,100—original $ 34,900—updated	$ 29,600—original
Ladabaum *et* *al*. [31] & Wang *et* *al*. [33]	USA			$ 38,700	$ 63,900
Barzi *et* *al*. [30]	USA			$ 46,900^	
**Age-Targeted Testing Strategies**					
Mvundura *et* *al*. [32]	USA	<50 years	No testing	$ 8,700	
		No limit	<50 years	$ 41,200	
Ladabaum *et* *al*. [31]	USA	≤50 years	No testing	$ 29,900	
		≤60 years	≤50 years	$ 36,200	
		≤70 years	≤60 years	$ 47,300	
		No limit	≤70 years	$ 94,900	
Sie *et* *al*. [34]	Netherlands	≤70 years	≤50 years	Dominant (cost-saving)	
Snowsill *et* *al*. [29,35]	UK	<50 years	No testing		$ 8,400
		<60 years	No testing		$ 11,800
		<70 years	No testing		$ 16,600
**Age and Family History-Based Testing**					
Ladabaum *et* *al*. [31]	USA	MMRpro	No testing	$ 32,700	
		Universal	MMRpro	$ 125,200	
Severin *et* *al*. [14]	Germany	RBG	No testing	$ 106,100	
		Universal	RBG	$ 347,700	
Barzi *et* *al*. [30]	USA	MMRpro	No testing	$ 35,100 ^	
		Universal	MMRPro	$ 144,100 ^	

^ As reported in Barzi *et* *al*., which did not state the year or years of the cost assumptions. ICER: incremental cost-effectiveness ratio; LY: life-years; QALY: quality-adjusted life-years; RBG: Revised Bethesda Guidelines criteria; MMRPpro software in the CancerGene software package [15].

Differences in estimates of health outcomes associated with identification of relatives with LS are of particular importance. Mvundura *et* *al*. projected 1.07 discounted LYs per relative identified with LS [32]; the revised CDC model [27] assumed 0.71 discounted LYs per relative. Ladabaum *et* *al*. projected 0.49–0.51 discounted LYs per relative with LS assuming incomplete uptake of testing and adherence; with complete identification and adherence the saving would be 0.84–0.88 discounted LYs per relative [31]. Severin *et* *al*. projected 0.49–0.58 discounted LYs per relative identified with LS [14]. Barzi *et* *al*. reported 0.59 discounted LYs per LS diagnosis by universal testing *vs*. no testing [30]. The factors contributing to these differences are itemized in section 3.3.3, section 3.3.4, section 3.3.5 and section 3.3.6.

#### 3.3.1. Testing Costs

Universal testing of newly diagnosed patients with CRC can be done in more than one way [20]. One way is to have pathology departments routinely test tumors using either IHC or MSI based on general surgical consent and then contact patients who test positive to offer genetic counseling for further testing. Another way is to contact patients around the time of CRC surgery and offer IHC and/or MSI testing of tumor tissue, followed by sequencing of mismatch repair (MMR) genes to identify a causative mutation for LS if the initial test results are positive. Some studies also assessed universal gene sequencing in patients with CRC. The CEA studies differed with regard to whether they assumed routine tumor testing with patient consent obtained only for germline testing [31,33,34] or that informed consent would be obtained prior to tumor as well as germline testing [14,29,32].

**Table 3:** Base case values of cost assumptions of routine testing for Lynch Syndrome in patients with colorectal cancer (CRC) and first-degree relatives, in 2014 US dollars.

Study	Pre-Test Counseling for CRC Patients	IHC	Post-Test Counseling	Counseling for Gene Sequencing	Gene Sequencing for *MLH1* Gene	Approaching and Counseling Relatives	Test for Known Family Mutation	Combined Cost of Counseling and Testing A Relative
Mvundura *et* *al*. [32]	22	290	106	194	899	156 * plus 194	61	411
Ladabaum *et* *al*. [31]	NR	300	112	198	942	118	492	610
Sie *et* *al*. [34]	25	184	136	0	1184	77	353	430
Snowsill *et* *al*. [29,35]	0	366	0	103	714	103	265	368
Severin *et* *al*. [14]	57	166	161	0	5268	57	281	338
Barzi *et* *al*. [30]	NR	300	112	198	942	118	492	610

* This cost estimate is based on the CDC model, which adjusted the estimate in the published article for inflation to 2007 dollars. IHC: immunohistochemistry; *MLH1*: mutL homolog 1; NR: Not reported.

Unit costs are reported in Table 3 for three tests: IHC (as an example of initial tests), MMR gene sequencing for CRC patients based on initial testing results, and targeted mutation analysis in relatives to test for the same mutation found in probands. Most cost assumptions were broadly similar. One exception is the cost to test for a single known mutation in the CDC study; Mvundura *et* *al*. based their estimate on cost accounting at a nonprofit laboratory and noted that the median price charged by laboratories was eight times the estimated cost [32]. In contrast, the CDC estimate of the combined cost of locating and approaching relatives and providing initial genetic counseling was more than three times higher than assumed in the other studies (Table 3). These differences do not have a large impact on the ICERs, as the combined cost estimates for counseling and testing relatives are similar across studies. Raising the combined cost in the CDC model to that of the UCSF model would raise the ICER in the CDC study by less than 5%.

Severin *et* *al*. reported the German reimbursement rate for gene sequencing to be approximately US $ 5,300, [14] which is 5–7 times higher than in other studies. This parameter is influential; when the higher figure was used in the CDC model, the ICER was doubled. Since the cost of testing for a single known mutation in the German study was intermediate between the UK and Netherlands estimates, one can assume that the cost of molecular genetic testing in general is not higher in Germany compared with other countries. It is the German reimbursement for gene sequencing alone that is an outlier.

#### 3.3.2. Surveillance Costs

The total cost of intensive colonoscopic surveillance is a function of the frequency at which colonoscopies are assumed to occur with and without knowledge of LS status, the unit cost of colonoscopies, the probabilities of serious complications (perforation or bleeding), and the unit cost of treating complications (Table 4). The majority of studies assumed that the cost of a colonoscopy is close to $ 700, but two studies assumed much lower costs [14,34]. Because complications are rare, differences in assumptions across studies, including leaving out complications, have little influence on ICERs of testing for LS.

**Table 4:** Cost estimates in cost-effectiveness studies of routine testing for Lynch Syndrome in patients with colorectal cancer, in 2014 US dollars.

Study	Direct Cost of Colonoscopy	Cost of Perforation	Cost of Bleeding	Complication Cost per Colonoscopy
Mvundura *et* *al*. [32] *	1043	19,471	6530	43
Ladabaum *et* *al*. [31] & Wang *et* *al*. [33]	690	11,025	6653	20
Sie *et* *al*. [34]	206	Not reported	Not reported	Not reported
Snowsill *et* *al*. [29,35]	911	7898	585	3
Severin *et* *al*. [14]	265	7555	3923	3
Barzi *et* *al*. [30]	690	11,025	6653	20

* The cost estimates are adjusted for inflation from those in the spreadsheet model. The relevant cost estimates in Table A1 were expressed in 1998 values.

As described in Table 1, three studies assumed that mutation carriers who choose to undergo surveillance would have colonoscopies every two years [29,32,34]. The remaining four studies assumed that it would be done each year [14,30,31,33]. Although it is known that colonoscopy every 1–2 years is superior to every 2–3 years [38], no conclusive evidence exists that annual testing is superior to biennial testing. The four studies that assumed biennial colonoscopy all had lower ICERs than the studies that assumed annual colonoscopies. Within the revised CDC model, doubling the unit cost of a colonoscopy would raise the ICER from $ 34,900 to $ 43,800 per LY.

#### 3.3.3. Cascade Testing of Relatives

Table 5 lists assumptions regarding cascade testing of family members: the numbers of relatives per proband who are tested, the probability a tested relative has the same mutation as the proband, the uptake of recommended prevention strategies among mutation carriers, the percent reduction in CRC risk among adherent mutation carriers, and the baseline risk of CRC among mutation carriers without intensive surveillance.

**Table 5:** Base case values of epidemiologic parameters in cost-effectiveness studies of routine testing for Lynch Syndrome (LS) in patients with colorectal cancer (CRC) that relate to asymptomatic mutation carriers.

Study	# Relatives Tested per Proband	% Relatives Testing Positive for Mutation	Uptake of Prevention among Mutation Carriers	Reduction in risk of CRC with LS Surveillance	Weighted Incidence of First CRC in Absence of Adherence to Prevention	Difference in Case-Fatality Rate of CRC in LS Relative to Non-LS Patients in Absence of Prevention
Mvundura *et* *al*. [32]	2.1	45%	79%	62%	41.3%—unadjusted	24%
Ladabaum *et* *al*. [31] & Wang *et* *al*. [33]	4	50%	80%	58%	46%–54% by age 70	25%–30%
Sie *et* *al*. [34]	8	39%	88%	63%	3.5% per year	NR
Snowsill *et* *al*. [29,35]	2.1	44%	80%	61%	43.5%–46.4%	21%
Severin *et* *al*. [14]	1.1	50%	81.8%	52%	42%—unadjusted 35.6%—adjusted	33%
Barzi *et* *al*. [30]	2.6	Not stated	60%–80%	56%	46%–54% by age 70	NR

NR: Not reported.

The number of relatives tested per proband is an influential parameter. The Dutch study that assumed eight relatives tested per proband assumed the most favorable economic outcomes, [34] whereas the least favorable (highest ICER) estimates came from a German study that assumed just 1.1 at-risk relatives (*i*.*e*., FDRs of identified mutation carriers as well as FDRs of probands) tested per proband [14]. If the number of at-risk relatives tested per proband in the revised CDC model [27] were cut from four to two (*i*.*e*., 1.04 instead of 2.08 relatives tested per proband), the ICER would increase from $ 34,900 to $ 62,600 per LY.

#### 3.3.4. Colorectal Cancer Epidemiology in Lynch Syndrome

Estimates of the lifetime incidence of CRC in the absence of prevention measures are potentially important because a lower baseline risk of cancer implies fewer deaths that could be avoided by prevention. Three studies assumed a weighted average of 41%–46% cumulative lifetime incidence of CRC among mutation carriers who survive to at least age 70 [14,29,32] and three other studies assumed somewhat higher incidence of 46%–54% [30,31,33]. The Dutch Radboud study assumed an extremely high annual incidence of CRC of 3.5% [34], which likely explains their conclusion that testing for LS would result in lower total costs; the narrow range of assumptions in the remaining studies contribute little to differences in ICERs.

The age distribution of incident CRC cases among persons with LS is often not documented. Severin *et* *al*. cited a published French study according to which the incidence of CRC is shifted to older ages [14,39]. Grosse *et* *al*. revised the CDC model to substitute the age-specific rates from the French study and found that the gain in LYs was reduced by 12% because the distribution of cases and deaths was shifted to older ages with shorter remaining life expectancy [27]. The impact on ICERs of differences in assumed age distributions in the remaining studies could not be assessed.

The risk of death from CRC, or case-fatality rate (CFR), among patients with LS relative to patients with sporadic CRC reflects assumptions about the staging of cancer. Two studies did not document assumptions about relative mortality among CRC patients [30,34]. Two studies modeled the CFR in patients with LS relative to other CRC patients as a constant, lower by 25%–30% [31] or 33% [14]. Two other studies modeled stage-specific relative risk of death, with weighted averages lower by 21% [29] or 24% [32], a modest difference in assumptions. However, Mvundura *et* *al*. [32] did not implement the stated assumptions, as noted by Severin *et* *al*. [14]. In the corrected CDC model, the estimated gains in LYs by diagnosis of LS were lower by 11% relative to the original published estimates [27].

#### 3.3.5. Effectiveness of Early CRC Detection and Surveillance

The effectiveness of intensive surveillance (annual or biennial colonoscopy beginning at age 20 or 25) in preventing incident CRC in asymptomatic mutation carriers was assumed to be 58%–63% in all studies, which indicates that this is not an important source of differential cost-effectiveness estimates. Severin *et* *al*. stated in their text that they assumed 58% reduction in incidence [14]. However, the actual percent reduction in cumulative CRC risk in their model was 52%, which is the relative difference between 35.5% risk without surveillance and 17.0% risk with surveillance.

#### 3.3.6. Family History-Based Testing

Three studies calculated ICERs for targeted testing based on criteria or models that include both age and family history. Severin *et* *al*. assumed that applying RBG criteria would detect 88% as many patients with LS as would universal offer of tumor testing [14]. The authors cited an international research study that reported that 88.1% of probands with LS who could be evaluated using the RBG criteria were detected using those criteria [13]. However, the authors of the latter study reported that among all probands, 68.6% fulfilled at least 1 RBG criterion, including age <50. Many patients could not be evaluated because of insufficient family history information. If the 68.6% figure were used instead, the incremental proportion of cases detectable through universal testing would have been almost three times higher, 31.4% instead of 11.9%, and the ICER for universal testing would have been 3 times lower.

Ladabaum *et* *al*. and Barzi *et* *al*. modeled five different predictive models that rely on detailed family history data: Amsterdam, RBG, and three statistical models—PREMM, MMRpro, and MMRpredict [15,30,31]. Both calculated that use of MMRpro would result in the lowest ICER. However, Barzi *et* *al*. noted that the MMRpro was also the most intensive and difficult predictive model to apply; they questioned whether the model would be feasible in routine clinical practice [30]. The MMRpro model requires information for each first- and second-degree relative on the age at diagnosis of colorectal cancer, age at diagnosis of endometrial cancer, and current age or age at last follow-up for those unaffected by CRC or EC [15].

#### 3.3.7. Health Utilities

Three studies reported estimates of QALY gains. In one of those studies, the calculation of QALYs was done as a sensitivity analysis and primarily reflected population-level mean health utilities, which decreases with increasing age [32]. Mvundura *et* *al*. also modeled a transient effect of CRC on health utilities, with a decrement of 0.1 assumed to last two years consistent with previous research [40,41]. The study assumed no decrement associated with testing for MMR mutations, citing expert opinion [42].

The UCSF study assumed a very large disutility of active cancer, a decrement of roughly 0.4 for up to five years [33]. In addition, Wang *et* *al*., assumed 12 months of disutility of roughly 0.3 from receiving a diagnosis of LS [33]. Another key assumption of the UCSF model is that FDRs experience substantial disutility (0.24–0.28) from learning that they are at elevated risk of CRC, independently of whether they choose to accept mutation testing or learn the results of the mutation analysis. Further, the authors assumed a disutility of 0.34 among newly diagnosed patients with CRC who decline to be tested for LS. These very large disutility estimates of the psychological impacts of testing for LS come from a time trade-off utility elicitation study [43]. Applying those estimates, Wang *et* *al*. reported a roughly 40% smaller gain in QALYs than LYs in their earlier publication [31,33]. That compares with a 15% difference in the estimates by Mvundura *et* *al*. [32].

The PenTAG review reviewed estimates of disutilities associated with cancer, surgeries, and LS [29]. The authors questioned the very high disutility estimates reported by Kupperman *et* *al*. [43] that were used by Wang *et* *al*. [33] as well as their duration, and adjusted them downwards in both magnitude and duration. Snowsill *et* *al*. [29] concluded from their systematic review that negative psychological effects of genetic testing are very small (0.00–0.04) and last no longer than four months. The UK model estimated discounted QALYs but not discounted LYs.

#### 3.3.8. Interventions beyond Colonoscopy Surveillance

Two studies modeled prophylactic surgery for female mutation carriers. Ladabaum *et* *al*. assumed that 18% of female mutation carriers undergo TAH/BSO surgery at age 40 and that this prevents endometrial and ovarian cancer [31]. Exclusion of TAH/BSO surgery from their model would reduce the gain in discounted life years by 37% and raise the ICER by 21% relative to the results that were reported. In the PenTAG model, the uptake of TAH/BSO surgery beginning at age 45 was assumed to be 55% for both probands and mutation carriers, but only prevention of endometrial cancer was modeled [29]. The authors concluded that excluding TAH/BSO would have little effect for female probands and mutation carriers, but would substantially reduce costs, thus leading to a lower ICER. Economic analyses specific to gynecologic cancers in LS have concluded that TAH/BSO is likely to be highly cost-effective [44,45,46].

The UCSF model modeled the costs of annual surveillance of female probands and mutation carriers for gynecologic cancers [31,33]. Because such testing was assumed to have no health benefits, the ICER was higher than it would have been if the costs of such surveillance had not been considered. To the extent that women with LS choose routine surveillance for gynecologic cancers, other CEA studies may have understated the costs associated with testing for LS.

Severin *et* *al*. reported that inclusion of aspirin prophylaxis made relatively little difference to survival or costs for individuals with LS, lowering the ICER of the preferred testing strategy by just 3% [14]. However, they assumed limited efficacy (37%), much lower than for colonoscopy, a limited duration of aspirin use (11 years), because they did not wish to extrapolate beyond the bounds of the CAPP2 trial data, and no substitution of aspirin for intensive surveillance. The efficacy among those who took either aspirin or placebo in the CAPP2 trial was approximately 60% [37]. A newly published registry study found that LS mutation carriers who took aspirin or ibuprofen for one month to five years had a 50% lower incidence of CRC and those who took it for more than five years had a 75% lower incidence [47]. However, that study did not report how many subjects followed intensive surveillance.

## 4. Conclusions

Is testing for LS in adults with CRC along with cascade testing of relatives cost-effective? That in large part is a function of projected effectiveness. The effectiveness of testing for LS, whether measured in discounted LYs or QALYs gained, is primarily a product of the reduction in CRC-associated mortality. No two articles reviewed calculated mortality reductions in quite the same way, which makes direct comparisons difficult. Cost-effectiveness is also a function of the comparator. Universal testing may appear cost-effective relative to no testing but not necessarily in comparison with selective or age-targeted testing strategies.

Whether testing is considered cost-effective may also depend on the meaning of “cost-effective.” Do decision-makers classify an intervention with an ICER above a single threshold value such as $ 50,000 as not cost-effective? Or, do they use a range of values, such as $ 50,000 to $ 100,000? How does the definition of cost-effective health intervention vary across countries? The World Health Organization suggests that interventions be considered “highly cost-effective” if the cost per disability-adjusted life-year or DALY is less than per capita GDP, and “cost effective” if the ICER is less than three times the per capita GDP [48]. For the United States, that approach implies a range of $ 53,000 to $ 160,000 in 2013 values.

A related question is whether the outcome metric is LYs or health-adjusted LYs. CEAs of cancer prevention strategies often use LYs because mortality benefits dominate quality of life impacts [32,49]. Because preventing deaths among adults results in fewer gains in QALYs than LYs, ICERs are higher when QALYs are used. However, QALYs are challenging to implement in LS due to lack of consensus on the disutilities associated with intermediate outcomes [29,33,43]. Two CEA studies assumed dramatically different magnitudes of negative psychosocial effects on mutation carriers of knowledge of LS, of genetic testing, and on other family members [29,33]. These differences had major implications for the calculation of QALYs and the estimated cost per QALY, which was much higher in the UCSF study which assumed large negative effects on carriers and family members [33]. The more conservative assumptions about psychosocial effects made in the PenTAG model [29] appear consistent with previously published literature as well as expert opinion.

Whether routine testing for LS in adults with newly diagnosed CRC is considered cost-effective depends on the assumed feasibility, cost, and sensitivity of selective testing using detailed family history data. One older modeling study concluded that universal testing would not be cost-effective in comparison with selective testing based on Bethesda criteria [50]. Two of the US studies reviewed here similarly suggest that universal testing does not appear cost-effective relative to selective testing, with an ICER > $ 100,000 per LY [30,31]. A German study reached qualitatively similar conclusions [14]. That presumes that such selective testing is feasible to implement in routine clinical practice, which is uncertain [51]. One US study reported that two-thirds of suspected cases of LS would not have been identified if the RBG criteria had been strictly followed [16]. No published study has demonstrated that testing based on complicated predictive models achieves close to 90% sensitivity in routine practice. One study of selective screening at the UCSF teaching hospital during 2007–2010 compared with universal testing conducted during 2010–2013 found that twice as many cases of LS were identified with universal testing [52]. CEA studies that include testing based on family history as a comparator should include the cost to obtain and interpret family history data [29,30,31].

Both universal tumor testing and testing just patients with CRC up to age 70, *i*.*e*., the Jerusalem criterion [24], have been implemented in large healthcare systems [53,54,55]. Ladabaum *et* *al*. calculated that the ICER for universal testing in that comparison was approximately $ 100,000 per LY [31]. Two CEA studies that excluded patients over age 70 both concluded that universal testing of patients up to age 70 would be highly cost-effective relative to no testing [14,34]. Other experts have also suggested that testing for LS using an age cutoff of 70 would be more cost-effective than universal testing. One commentary argues that testing patients under age 70 would reduce testing costs by 49% while detecting 91% of cases of LS [56]. Another analysis calculated that an age cutoff of 70 would miss 14% of cases and reduce costs by 35% [57]. More work is needed to assess the incremental cost-effectiveness of universal testing relative to testing with an age cutoff of 70.

The analytical perspective and the methods for assessing the costs of clinical services also influence cost-effectiveness calculations. CEAs of testing for LS have not followed a consistent approach. Studies which stated that they followed a societal or healthcare sector perspective, both of which presume estimates of resource costs, for the most part relied on reported expenditures [30,32]. Just one study attempted to estimate resource costs for laboratory tests [32], but that study relied on 1998 Medicare payment rates adjusted for inflation; the actual cost of colonoscopy is likely to have been lower. For analyses from the payer perspective [14,29,31,33], it is the stakeholder expenditure that is pertinent, not resource costs [58].

One important factor is the number of relatives tested per proband, which varies across studies from just over 1 to 8. Within a model, the ICER is roughly proportional to the number of relatives tested per proband. For example, when the number of relatives tested per proband in the revised CDC model [27] is reduced by one-half from the baseline value, the ICER almost doubled from roughly $ 32,400 to roughly $ 56,200 per LY. Other authors have also noted that the cost-effectiveness of testing for LS may be contingent on the number of relatives tested and found to carry mutations [59]. Evidence from one US implementation study suggests that the assumption of 2.1 at-risk relatives tested per proband [29,32] might be conservative; Marquez *et* *al*. report that 13 relatives were tested for four probands identified at one hospital and six were found to be mutation carriers [55]. Larger studies are needed with data from diverse health institutions to produce reliable estimates.

This review is not exhaustive. It does not address the efficiency and costs of different tumor testing strategies, such as IHC first *vs*. MSI first. The cost per case detected has been modeled in other studies in addition to the CEAs included in this review [19,60,61,62,63,64]. The implications of use of preventive strategies for endometrial and ovarian cancer in females with LS require further investigation. Future studies of the cost-effectiveness of LS testing might model the effectiveness of colonoscopy and aspirin preventive strategies on the basis of observational data among LS mutation carriers regarding the use of just one or both preventive strategies. An important question that still needs to be addressed is whether long-term prophylactic use of aspirin by mutation carriers might make LS testing more cost-effective.

Finally, this paper does not address the controversial proposal to offer direct gene sequencing testing for LS in primary care patients, as proposed in a commercially-produced CEA modeling study [65]. That analysis assumed that detailed, fully accurate family history data would be available cost-free to primary care providers, which is implausible [51,66]. A recent modeling study concluded that such testing is not cost-effective under plausible assumptions [30].

The diversity in assumptions and estimates in this review preclude drawing conclusions about whether universal testing for LS is or is not cost-effective in absolute terms. Testing either all CRC patients for LS or patients up to age 70 years *vs*. no testing is likely to be considered cost-effective in the United States. Conclusions in other countries may vary depending on ICER thresholds and reimbursement rates for genetic testing and counseling. Cost-effectiveness of testing is contingent on systems of care in which relatives are counseled and offered genetic testing that is reimbursed by payers. Further, demonstration of actual cost-effectiveness requires documentation from routine clinical practice (not research protocols) of numbers of diagnoses of LS among relatives and uptake of intensive surveillance for CRC. A more challenging question is to identify the optimal testing strategy. We need real-world data on the sensitivity and costs of different testing strategies, including universal testing, age-targeted testing using different age cutoffs, and use of predictive models, including detailed family history data.

This case study has implications for the economic evaluation of genomic testing applications in general. First, context matters. Testing costs and uptake of genomic testing vary across populations and healthcare systems. Strategies to improve the uptake of counseling and testing may be crucial to improving the effectiveness and cost-effectiveness of genomic testing. Second, the cost-effectiveness of a testing strategy depends on the alternative to which is it compared. Universal testing for a condition for which highly effective prevention strategies are available is likely to prove cost-effective in many settings compared with no testing but may not be cost-effective in comparison with selective testing based on predictive models or criteria. The accuracy and cost of selective testing strategies should be documented in real-world settings before drawing conclusions about cost-effectiveness.
